# Remarkable Response to Dabrafenib and Trametinib in a V-Raf Murine Sarcoma Viral Oncogene Homolog B (BRAF) V600E-Mutated Lung Adenocarcinoma Patient With Poor Performance Status and Respiratory Failure: A Case Report

**DOI:** 10.7759/cureus.75217

**Published:** 2024-12-06

**Authors:** Satoshi Anai, Kohei Isa, Rin Chibana, Shiho Ueno, Yoko Sato

**Affiliations:** 1 Division of Respiratory Medicine, Yuuai Medical Center, Okinawa, JPN

**Keywords:** braf v600e, dabrafenib plus trametinib, non-small cell lung cancer (nsclc), poor performance status, respiratory failure

## Abstract

We report the case of a 73-year-old man with progressive dyspnea and acute respiratory failure. Imaging revealed extensive infiltrative shadows in the right lung. A bronchoscopic biopsy confirmed primary lung adenocarcinoma harboring the BRAF V600E mutation. The patient's performance score was three, requiring four liter/minute of supplemental oxygen to maintain an oxygen saturation of 90%. After a thorough discussion and informed consent, treatment with dabrafenib and trametinib was initiated despite the risks associated with his condition. Following the initiation of therapy, the patient exhibited significant clinical and radiological improvements. He continues to receive the combination therapy without significant adverse events, and his disease remains stable. We report this as the first case that demonstrates dabrafenib and trametinib can be effective and well-tolerated in patients with BRAF V600E mutated non-small cell lung cancer (NSCLC), even in those with poor performance status and acute respiratory failure. This result provides early evidence supporting the cautious use of molecular-targeted therapies in such patients.

## Introduction

The guidelines state that tyrosine kinase inhibitors should be considered in patients with advanced non-small cell lung cancer (NSCLC) with driver gene mutations and poor performance status (PS three or four), with attention paid to the toxicity profile [1]. Eastern Cooperative Oncology Group (ECOG) PS three refers to a state in which a patient can perform only limited self-care and spends more than 50% of the day in bed or in a chair. Notably, the effectiveness of molecular-targeted therapies for epidermal growth factor receptor (EGFR) gene mutations or anaplastic lymphoma kinase (ALK) gene rearrangements in lung cancer patients with low PS has been reported in clinical trials [2,3]. However, there are no reports of clinical trials for cases with poor PS in v-Raf murine sarcoma viral oncogene homolog B (BRAF) gene mutated lung cancer. In a case report, it was reported that an elderly patient with BRAF V600E-mutated lung cancer and poor PS died within a short time after starting treatment [4]. Therefore, careful consideration is necessary when treating similar patients. In our case report, we present the first case of a patient with BRAF V600E-mutated lung cancer, poor PS, acute respiratory failure, and a history of treatment for castration-resistant prostate cancer who was administered dabrafenib and trametinib. After initiating treatment, the patient demonstrated significant tumor shrinkage, improved PS, and an improved respiratory status.

## Case presentation

A 73-year-old man with a history of smoking 20 cigarettes per day for 30 years visited the emergency room complaining of progressive dyspnea. Five months before visiting the emergency room, he had experienced a gradually worsening sense of dyspnea. Two days prior to admission, he experienced a brief loss of consciousness lasting a few seconds following severe exertional dyspnea.

As a part of his medical history, ten years ago, he was diagnosed with castration-resistant prostate cancer with multiple bone metastases at a local general hospital. At the time of diagnosis, his prostate-specific antigen (PSA) level was 119 ng/mL, and his Gleason score was nine. He was started on bicalutamide and degarelix combination therapy for prostate cancer. After one and half years of treatment, bicalutamide was discontinued. Seven years ago, he was referred to our urology department and continued receiving degarelix. Three years ago, he switched from degarelix to goserelin.

**Figure 1:**
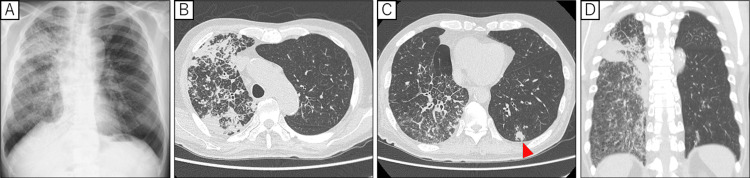
Pre-treatment chest X-ray and CT images showing tumor regression (A) Chest X-ray showed an infiltrative shadow mainly in the right lung field. (B-D) Chest CT showed an infiltrative shadow with thickening of the interlobular septa in the right lung, as well as ground-glass opacity. (C) In addition, a nodule* thought to be an intrapulmonary metastasis was observed in the lower lobe of the left lung (*Intrapulmonary metastasis marked by the red arrowhead).

On examination, his oxygen saturation was 88% on room air. Vital signs included blood pressure of 80/46 mmHg, heart rate of 95 beats per minute, body temperature of 36.7°C, and respiratory rate of 24 breaths per minute. Physical examination revealed slight moist rales on inspiration in the right lung. Chest X-ray (CXR) (Figure 1A) showed an infiltrative shadow encompassing the entire right lung.

A computed tomography (CT) scan of the chest revealed infiltrative shadows and ground-glass opacities in the right lung, accompanied by thickening of the interlobular septa (Figures 1B-1D). Additionally, a small nodule was seen in the lower lobe of the left lung (Figure 1C).

Laboratory tests showed a slight increase in white blood cell count (8,500/μL) and C-reactive protein (2.25 mg/dL) (Table 1).

**Table 1:** Patient's laboratory test results WBC: White blood cell, Hb: Hemoglobin, Plt: Platelet, TP: Total protein, Alb: Albumin, AST: Aspartate aminotransferase, ALT: Alanine aminotransferase, LDH: Lactate dehydrogenase, BUN: Blood urea nitrogen, Cre: Creatinine, UA: Uric acid, Na: Serum sodium concentration, Cl: Serum chloride concentration, K: Serum potassium concentration, CRP: C-reactive protein, CEA: Carcinoembryonic antigen, PSA: Prostate specific antigen.

Parameter (units)	Results	Reference value
WBC (x10^3^/μL)	8.5	3.3-8.6
Hb (g/dL)	14.3	13.7-16.8
Plt (x10^4^/μL)	24.3	15.8-34.8
TP (g/dL)	6.9	6.6-8.1
Alb (g/dL)	3.7	4.1-5.1
AST (IU/L)	19	13-30
ALT (IU/L)	11	10-42
LDH (IU/L)	234	124-222
BUN (mg/dL)	27	8-20
Cre (mg/dL)	1.46	0.65-1.07
UA (mg/dL)	9.8	3.7-7.8
Na (mmol/l)	137	138-145
Cl (mmol/l)	101	101-108
K (mmol/l)	3.8	3.6-4.8
CRP (mg/dL)	2.25	0.00-0.14
CEA (ng/mL)	23.4	<5.0
PSA (ng/mL)	<0.008	<4.0

**Figure 2:**
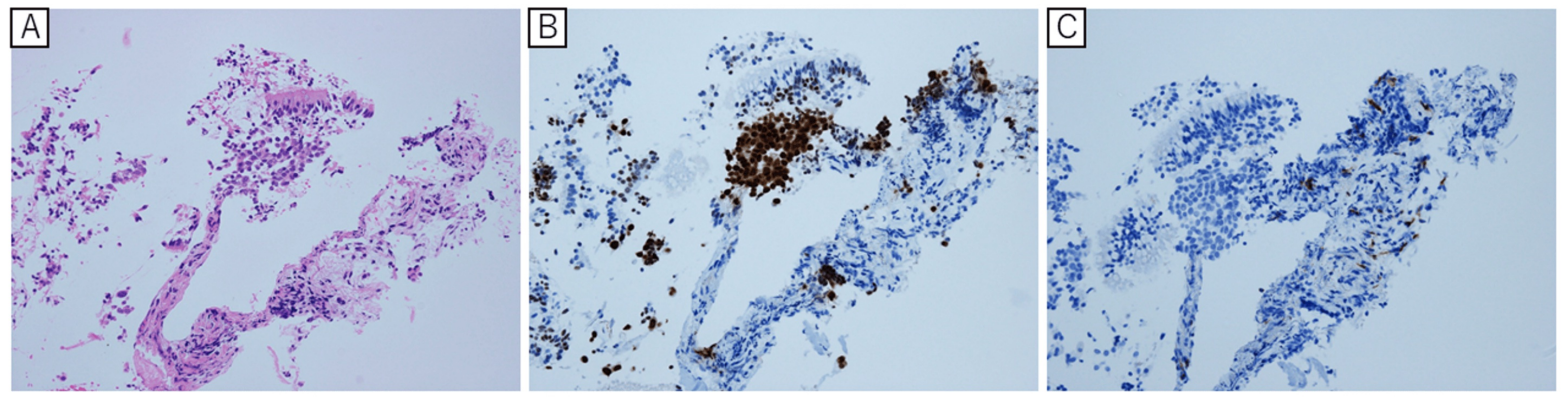
Histopathological images from bronchoscopic lung biopsy (A) Pathological diagnosis of adenocarcinoma was based on the presence of atypical cells with scattered nuclei and no cilia in the fragmentary tissue (HE staining, original magnification, ×200). Immunostaining showed (B) TTF1 positivity (original magnification, ×200) and (C) PSA negativity (original magnification, ×200). H&E: Hematoxylin and eosin, TTF1: thyroid transcription factor-1, PSA: prostate-specific antigen

There was no increase in PSA (<0.008 ng/mL), but carcinoembryonic antigen (CEA) was elevated at 23.4 ng/mL. Based on his clinical presentation, differential diagnoses included bacterial pneumonia, lung metastasis of prostate cancer, primary lung cancer, and drug-induced pneumonia caused by goserelin. The patient was admitted to the respiratory medicine department for further evaluation and treatment. After hospitalization, his general condition was assessed as PS three due to respiratory failure. He required four liters/minute of oxygen to maintain an oxygen saturation of 90%. He was administered ceftriaxone (2 gms/day) for seven days and azithromycin 500 mg intravenously for three days. Despite antibiotic therapy, there was no improvement in the radiographic findings. The absence of fever and the lack of respiratory improvement suggested a low likelihood of bacterial pneumonia. Therefore, antibiotics were discontinued after a total of seven days. Sputum cytology performed during hospitalization detected adenocarcinoma cells. Consequently, a bronchoscopic biopsy was conducted. Histopathological examination confirmed adenocarcinoma (Figure 2A). Immunohistochemical staining showed positive thyroid transcription factor-1 (TTF-1) (Figure 2B) and negative PSA (Figure 2C), suggesting primary lung adenocarcinoma [5].

Further molecular analysis using the AMOY 9-in-1 kit (Amoy Diagnostics Co. Ltd., Xiamen, China) revealed a BRAF V600E mutation. Programmed death-ligand 1 (PD-L1) expression was 50% (tumor proportion score). Tumor lesions were identified in the upper, middle, and lower lobes of the right lung, and nodules suspected to be tumor lesions were also found in the lower lobe of the left lung (Figures 1B-1D). No intracranial lesions were observed on head imaging. Based on these findings, the patient was diagnosed with right lung adenocarcinoma, cT4N0M1a, Stage IVA, harboring a BRAF V600E mutation. Given the patient's general condition of PS three and the presence of respiratory failure, the use of anticancer agents, including molecular-targeted therapies, carried a very high risk of worsening the prognosis due to potential complications such as drug-induced pneumonitis. After thoroughly explaining the risks to the patient and his family, they agreed to proceed with treatment. On the 26th day of hospitalization, treatment with dabrafenib (150 mg twice daily) and trametinib (2 mg daily) was started as the standard treatment dose. Following the initiation of therapy, gradual improvements in oxygenation were observed. By the seventh day of treatment, the patient no longer required supplemental oxygen.

**Figure 3:**
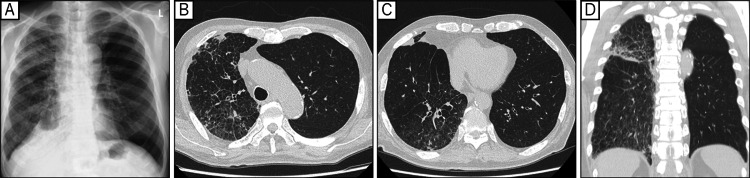
Post-treatment chest X-ray and CT images showing tumor regression (A-D) After starting dabrafenib+trametinib, the infiltrative shadow and ground-glass opacity due to lung cancer showed marked improvement on imaging.

After 10 days of treatment, CXR also showed improvement of the pulmonary infiltrate in the right lung field (Figure 3A). The patient was discharged on the 15th day after initiating treatment. A CT scan obtained six weeks after initiating treatment showed marked improvement of the pulmonary infiltrate. (Figures 3B-3D). He continues to receive dabrafenib and trametinib without significant adverse events, and the tumor has remained stable.

## Discussion

This case represents the first report of a patient with poor PS and acute respiratory failure who was administered dabrafenib and trametinib and achieved a significant response without experiencing any serious side effects. BRAF V600E mutation is the most common BRAF mutation, occurring in one to two percent of NSCLC cases, and is more prevalent in men and smokers [6,7]. In a phase II study involving patients with advanced NSCLC harboring BRAF V600E mutation and PS less than two, the combination of dabrafenib and trametinib demonstrated an overall response rate of 64% in patients receiving first-line treatment. Additionally, the median progression-free survival was 10.9 months, and the median overall survival was 24.6 months [7,8]. The reported adverse events include fever, nausea, fatigue, and cardiac dysfunction, necessitating careful monitoring. Evidence from clinical trials indicates that molecular-targeted treatments are effective for patients with poor PS resulting from EGFR mutations or ALK rearrangements [2,3]. We have also previously reported a case of metastatic ALK-positive lung cancer with poor PS and brain herniation, in which the patient responded remarkably to alectinib after brain tumor resection [9].

One reported case describes a metastatic NSCLC patient with poor performance status who had BRAF V600E mutation and developed severe pneumonia two weeks after initiating tyrosine kinase inhibitor therapy (dabrafenib and trametinib), ultimately resulting in death [4]. In that case, the patient's poor prognosis was partly attributed to the deterioration of PS from one to four over the four weeks between the initial hospital visit and the commencement of treatment. In contrast, to maintain our patient's general condition and enable prompt initiation of treatment, we implemented rehabilitation measures, promptly assessed his condition, performed bronchoscopy for rapid pathological confirmation, and utilized the AMOY 9-in-1 kit which has a shorter turnaround time (four to seven days) compared to Oncomine Dx target test (six to nine days) [10]. As a result, treatment was initiated 26 days after admission, and the patient's PS was maintained at three, which we believe contributed to the favorable treatment outcome. Furthermore, our patient had extensive tumorous lesions in the right lung, with imaging revealing cancerous lymphangitis and acute respiratory failure. The concurrent development of pneumonia could have significantly worsened the prognosis. However, even in patients with poor general condition, the presence of a driver gene mutation may offer a good chance of a favorable outcome with molecular-targeted therapy [2,3]. Therefore, it is essential to provide thorough explanations to patients and their families beforehand. In this case, we conducted repeated, careful discussions to ensure that the patient and his family fully understood the disease and the treatment options. Their informed consent allowed us to proceed with treatment, leading to a positive prognosis.

## Conclusions

This case demonstrates that a patient with poor PS and acute respiratory failure can achieve marked efficacy with dabrafenib and trametinib, resulting in tumor shrinkage, improved PS, and enhanced oxygenation. Given the rarity of BRAF V600E mutations, evaluating their effects through clinical trials is challenging. However, this result provides early evidence supporting the cautious use of molecular-targeted therapies in such patients.
